# RNA-Seq and Genome-Wide Association Studies Reveal Potential Genes for Rice Seed Shattering

**DOI:** 10.3390/ijms232314633

**Published:** 2022-11-23

**Authors:** Linxuan Wu, Jicheng Yue, Jiafeng Wang, Wenyu Lu, Ming Huang, Tao Guo, Hui Wang

**Affiliations:** National Engineering Research Center of Plant Space Breeding, South China Agricultural University, Guangzhou 510642, China

**Keywords:** rice, seed shattering, GWAS, transcriptome analysis, haplotype analysis

## Abstract

The loss of the shattering ability is one of the key events in rice domestication. The strength of the seed shattering ability is closely related to the harvest yield and the adaptability of modern mechanical harvesting methods. In this study, using a population of 587 natural rice cultivars, quantitative trait loci associated with seed shattering were detected by genome-wide association studies (GWASs). We consider the quantitative trait loci (QTLs) *qBTS1* and *qBTS3* to be the key loci for seed shattering in rice. Additionally, the abscission zone (AZ) and nonabscission zone (NAZ) of materials with a loss of shattering (DZ129) and easy shattering (W517) were subjected to RNA-Seq, and high-quality differential expression profiles were obtained. The AZ-specific differentially expressed genes (DEGs) of W517 were significantly enriched in plant hormone signal transduction, while the AZ-specific DEGs of DZ129 were enriched in phenylpropanoid biosynthesis. We identified candidate genes for the lignin-associated laccase precursor protein (*LOC_Os01g63180*) and the glycoside hydrolase family (*LOC_Os03g14210*) in the QTLs *qBTS1* (chromosome 1) and *qBTS3* (chromosome 3), respectively. In summary, our findings lay the foundation for the further cloning of *qBTS1* and *qBTS3*, which would provide new insights into seed shattering in rice.

## 1. Introduction

Loss of shattering is one of the key events in the rice domestication process. The initial selection may be unintentional because rice with less shattering is easier to harvest. So far, the shattering allele has been replaced during the domestication process [1]. Rice shattering is one of the most important agronomic traits in rice cultivation and breeding. The abscission layer (AL) in the rice abscission zone (AZ) is made up of one to two layers of small, rounded parenchyma cells, while the stem cells and glume cells located nearby are made up of large sclerenchyma cells [2]. The adjacent cell layers have thicker, lignified cell walls, which can provide the mechanical force needed for abscission [2,3,4].

Seed shattering is a complex trait regulated by many genes [5]. The seed shattering gene *SH4* cloned from indica rice is the main gene of rice seed shattering, encoding a Myb3 domain transcription factor, which affects the formation and development of AL and promotes the hydrolysis of AL cells during seed shattering [1,6]. *SHA1*, which is homologous to the *SH4* gene, does not affect AL formation; however, studies have shown that the g237t mutation results in the substitution of K79N (a lysine residue mutated to an asparagine residue), causing in a loss of seed shattering [6]. The *qSH1* gene, located on chromosome 1, is another major gene involved in rice shattering and encodes a BELL homeobox protein. A single nucleotide polymorphism (SNP) at 12 kb (G to T) in the 5′ regulatory region in *qSH1* deprives the shattering characteristic. The mutation resulted in the decreased expression of *qSH1* in spikelet abscission cells, causing an inability to form the AL and loss of seed shattering [5,7]. *SHAT1*, *OsCPL1*, and *SSH1* also regulate grain shattering in rice [8,9,10,11,12,13]. *SH5*, homologous to the *qSH1* gene, inhibits lignin deposition and regulates seed shattering in rice. Studies have demonstrated that *OSH15* interacts with *SH5* to inhibit lignin synthesis and enhance seed shattering [9,14]. These studies indicated that seed shattering depends on the development of an AZ and the deposition of lignin.

In recent years, genome-wide association studies (GWASs) based on SNP markers have become a primary method for quantitative trait mining in model crops such as rice [15,16,17]. By simultaneously screening many accessions, GWAS mapping makes it possible to identify the genetic variation underlying complex phenotypes [17]. Numerous studies have identified candidate genes and quantitative trait loci (QTLs) for agronomic parameters such as crop development and stress tolerance [15,16,18,19,20,21]. With advances in sequencing technologies and its decreasing costs, mutual analysis with GWAS and multiomics has become a popular approach for identifying loci for agronomic traits in rice. For example, candidate gene screening and prediction methods combine GWAS mapping and RNA-seq data [22,23,24].

However, only a few studies have used GWAS mapping to identify potential genes for rice seed shattering. In this study, we aimed to detect QTLs related to rice seed shattering using a GWAS-based approach. On chromosome 1, *qBTS1*, a significant QTL that influences seed shattering, was discovered. *LOC Os01g63180* emerged as the most promising candidate gene in *qBTS1* when the results of RNA-seq and the GWAS were combined. A similar technique was used to locate *LOC Os03g14210* on chromosome 3 as a potential gene in QTL *qBTS3*. The findings of this study have significant implications for cloning rice shattering genes and understanding associated molecular pathways.

## 2. Results

### 2.1. Phenotypic Evaluations

**Figure 1:**
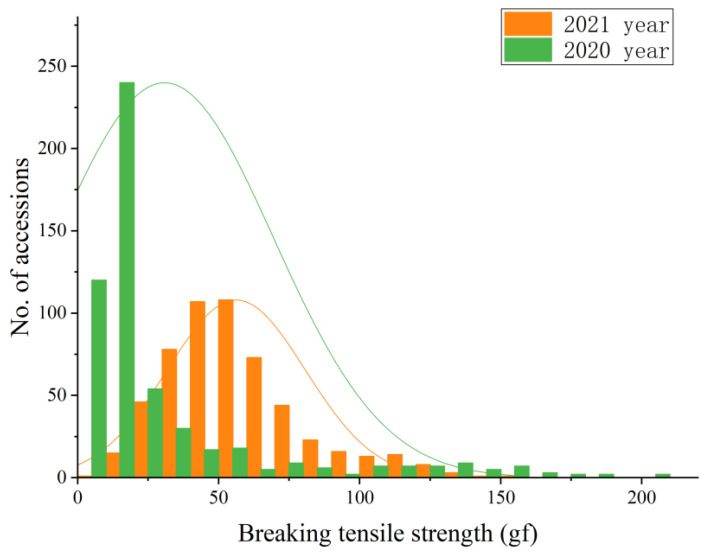
Phenotypic diversity of 587 rice varieties.

The phenotypic characteristics of 587 accessions were quantified. At 30 days after heading (DAH), we estimated the degree of seed shattering by measuring the tensile strength at break (or breaking tensile strength, BTS) values (Figure 1, Table S1). The average BTS value was 30.84 gf (1.72~206.48 gf) in the 2020 late season, and the average BTS value was 56.22 gf (8.3~153.6 gf) in the 2021 early season. The phenotypic data showed that the BTS values recorded in the early and late seasons had errors ranging from 0 to 50 gf.

### 2.2. Correlation Map or Rice Shattering Traits

**Figure 2:**
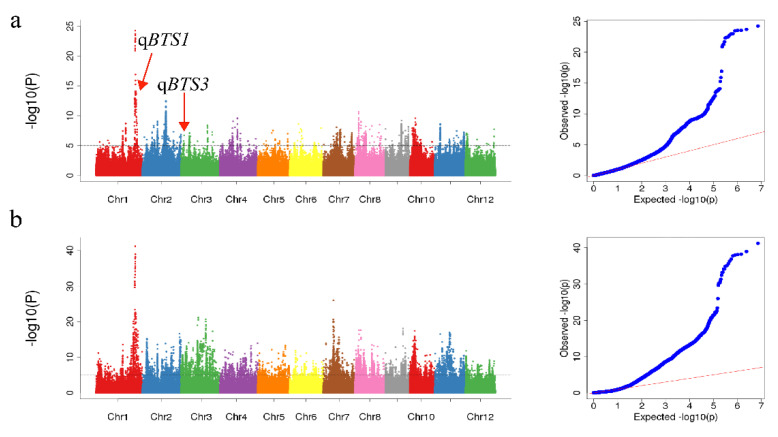
Manhattan and quantile–quantile (QQ) plots for rice seed shattering: (**a**) 2021 early season. (**b**) 2020 late season. The red lines represent the significant −log10(*p* value) (*p* = 1.0 × 10^−5^).

We conducted a GWAS of the BTS phenotypic values based on the produced high-density SNP marker data to discover novel loci associated with seed shattering. The Manhattan plots and quantile–quantile (QQ) plots are shown in Figure 2a (2021 early season) and Figure 2b (2020 late season). In this study, only associated loci that exceeded the *p* value threshold (-log10(*p* value) > 5) and had significant peak signal replicates in both datasets were considered. We detected 52 and 58 QTLs associated with seed shattering in 2021 early season and 2020 late season, respectively (Table S14). Significant peaks at chr01_36461792 and chr03_25163818 were located on chromosomes 1 and 3, respectively, which were consistent with the rice shattering genes *qSH1* and *OsYABBY2*, respectively [5,25]. The results demonstrate the accuracy of the GWAS results and have the potential to facilitate the exploration of other genes controlling seed-shattering traits. This study revealed two important SNPs, *qBTS1.1* (chr01 36637713) and *qBTS3.1* (chr03 7910523), located on chromosomes 1 and 3, respectively.

### 2.3. Identification and Histological Observation of Extreme Materials

**Figure 3:**
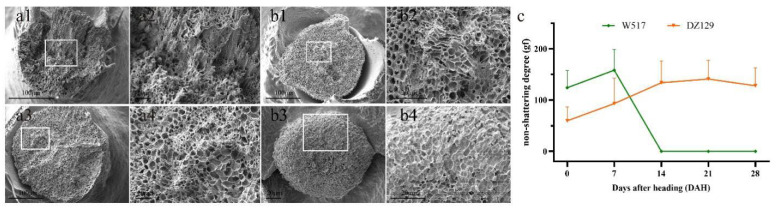
Scanning electron microscopy of tomographic sections of the rice AZ: (**a1**–**a4**) The tomographic SEM of DZ129’s AZ; the fault plane of (**a1**,**a2**) on 0 DAH, and the fault plane of (**a3**,**a4**) at 28 DAH. (**b1**–**b4**) The tomographic SEM of W517’s AZ; the fault plane of (**b1**,**b2**) on 0 DAH, and the fault plane of (**b3**,**b4**) on 14 DAH. Scale bars: a1, a3, and b1 are 100 μm; a2 and a4 are 10 μm; b2, b3, and b4 are 20 μm. (**c**) Comparison of DZ129 and W517 BTS values at 0, 7, 14, and 28 DAH. Values are means ± SDs (n = 40 grains).

Among the collected natural population materials, there were significant differences in the seed shattering of DZ129 and W517. Seed shattering was assessed using the BTS value. The BTS value of DZ129 first increased with DAH and then remained between 127 and 141 gf. In contrast, the BTS value of W517 first increased and then decreased, and the grain naturally shattered after 14 DAH (Figure 3c). Further SEM observations were made of the AZ on 0, 14, and 28 DAH. At 0 DAH, the AZ of W517 was relatively rough (Figure 3(b1,b2)), and, at 14 DAH, the AZ formed a completely smooth fracture surface (Figure 3(b3,b4)). At 28 DAH, the AZ of DZ129 was still rough (Figure 3(a1–a4)). The results showed that the formation and degradation of the abscission layer affected the degree of seed shattering. Materials that easily shattered formed a smooth fracture surface, while materials that lacked shattering did not form an abscission layer and had a rough fracture surface.

### 2.4. Differentially Expressed Genes between the AZ and NAZ of Extreme Materials

**Figure 4:**
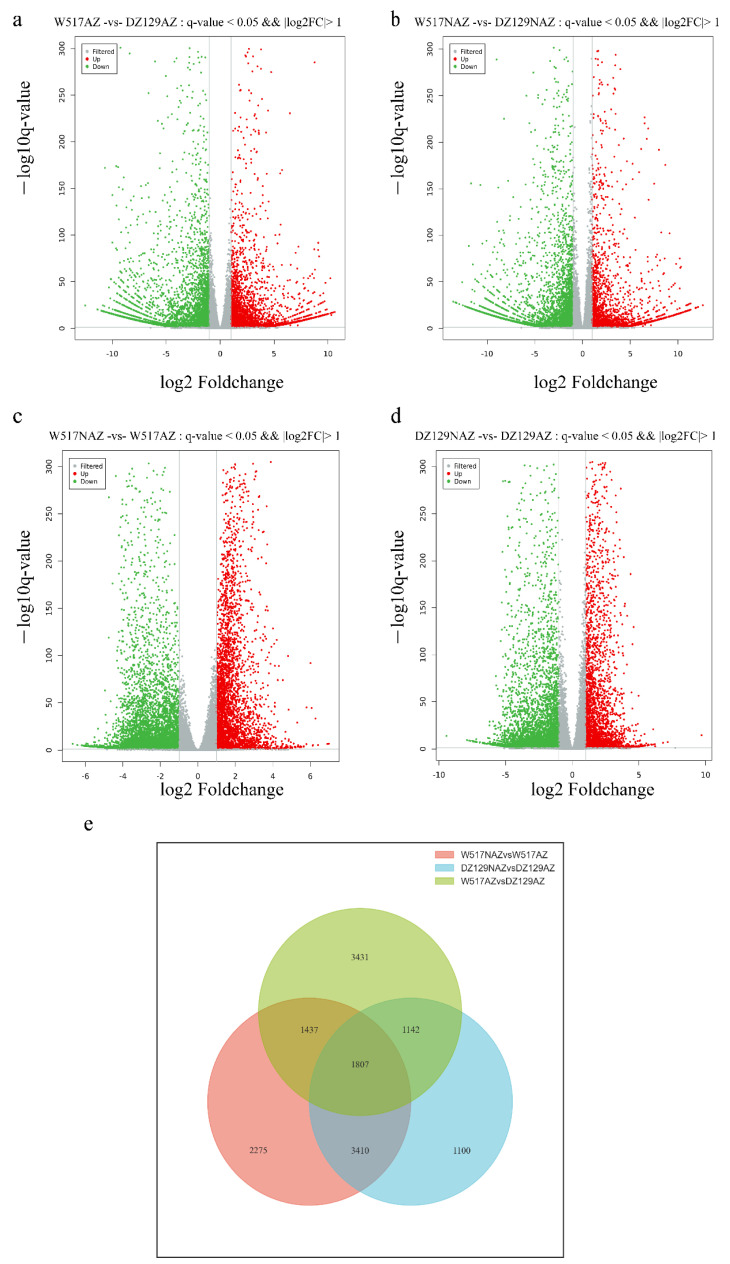
RNA-seq analysis between the AZ and NAZ of extreme materials: (**a**) Volcano plot displaying DEGs between the extreme materials at the AZ (W517AZ vs. DZ129AZ). (**b**) Volcano plot displaying DEGs between the extreme materials at the NAZ (W517NAZ vs. DZ129NAZ). (**c**) Volcano plot displaying DEGs between the AN and NAZ of W517 (W517NAZ vs. W517AZ). (**d**) Volcano plot displaying DEGs between the AZ and NAZ of DZ129 (DZ129NAZ vs. DZ129AZ). (**e**) Venn diagram analysis of the third set of DEGs.

To explore candidate genes associated with seed shattering, RNA was extracted from the AZ and NAZ of DZ129 and W517 at 10 DAH for RNA-seq. In W517AZ vs. DZ129AZ, we identified 7817 differentially expressed genes (DEGs) (3370 upregulated, 4447 downregulated) (Figure 4a); 7521 DEGs were identified in W517NAZ vs. DZ129NAZ (2868 upregulated, 4653 downregulated) (Figure 4b); 8929 DEGs were identified in W517NAZ vs. W517AZ (3610 upregulated, 5319 downregulated) (Figure 4c); 7459 DEGs were identified in DZ129NAZ vs. DZ129AZ (2838 upregulated, 4621 downregulated) (Figure 4d). A large number of DEGs were identified between the extreme materials, with more downregulated genes than upregulated genes; however, not all of these genes were associated with seed shattering. Therefore, we divided all of these genes into three groups: the AZ-specific DEGs of W517 (the intersecting genes for W517NAZ vs. W517AZ and W517AZ vs. DZ129AZ with DEGs overlapping with those for DZ129NAZ vs. AZ excluded), with a total of 1437 DEGs (Figure 4e, Table S3); the AZ-specific DEGs of DZ129 (the intersecting genes for DZ129NAZ vs. DZ129AZ and W517AZ vs. DZ129AZ with DEGs overlapping with those for W517NAZ vs. W517AZ excluded), with a total of 1142 DEGs (Figure 4e, Table S4); and the core differential genes (DEGs overlapping for W517NAZ vs. W517AZ, W517AZ vs. DZ129AZ, and DZ129NAZ vs. DZ129AZ), for a total of 1807 DEGs (Figure 4e, Table S5).

### 2.5. GO and KEGG Enrichment Analysis of DEGs

**Figure 5:**
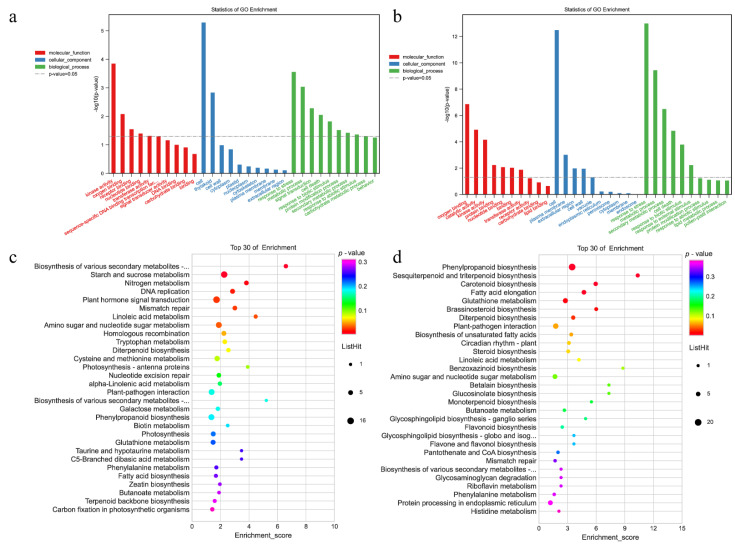
Gene ontology (GO) and Kyoto Encyclopedia of Genes and Genomes (KEGG) enrichment analysis: (**a**) GO analysis of the AZ-specific DEGs of W517. (**b**) GO analysis of the AZ-specific DEGs of DZ129. (**c**) KEGG analysis of the AZ-specific DEGs of W517. (**d**) KEGG analysis of the AZ-specific DEGs of DZ129.

To investigate DEG functions, gene ontology (GO) enrichment analysis of the AZ was performed on W517- and DZ129-specific DEGs. In total, 1437 and 1142 AZ-specific DEGs were assigned to 90 and 85 GO terms in W517 (Figure 5a) and DZ129 (Figure 5b), respectively. Within the cellular components category, the AZ-specific DEGs of W517 were enriched in cell, thylakoid, and cell wall; however, cell, plasma membrane, and extracellular region were the most abundant terms for the AZ-specific DEGs of DZ129. Additionally, for the AZ-specific DEGs of W517, signal transduction was significantly enriched in the biological process category, while it was not enriched for the AZ-specific DEGs of DZ129. However, for catalytic activity in the molecular function category, the AZ-specific DEGs of DZ129 and W517 showed the opposite enrichment result.

To further identify complex biological behaviors in the transcriptome and explore the biological functions of DEGs, Kyoto Encyclopedia of Genes and Genomes (KEGG) enrichment analysis was performed on the DEGs. The most representative pathways for the discovery of AZ-specific DEGs in W517 were the biosynthesis of various secondary metabolites-part 3, starch and sucrose metabolism, nitrogen metabolism, DNA replication, and plant hormone signal transduction. The most representative pathways for the discovery of the AZ-specific DEGs of DZ129 were phenylpropanoid biosynthesis, sesquiterpenoid and triterpenoid biosynthesis, carotenoid biosynthesis, fatty acid elongation, and glutathione metabolism. Interestingly, the AZ-specific DEGs of W517 and DZ129 were significantly enriched in nonoverlapping pathways (top five), revealing that the degree of shattering difficulty in rice is regulated by different pathways. KEGG analysis of the transcriptome data showed that the biosynthesis of various secondary metabolites-part 3, plant hormone signal transduction, and other pathways were related to the easy shattering of rice. Meanwhile, the significant enrichment of phenylpropane biosynthesis indicated that the deposition of lignin resulted in the loss of seed shattering.

Among the core DEGs, the degree of the regulation of seed shattering is crucial. The key to regulating the growth and development of the AZ is to control the degree of seed shattering. Further in-depth analysis of the core DEG set was carried out to examine DEGs with opposite regulation patterns in W517NAZ vs. AZ and DZ129NAZ vs. AZ or the same regulation patterns but with log_2_ FC (W517NAZ vs. AZ)/log_2_ FC (DZ129NAZ vs. AZ) values ≥ 1, which were considered to be the core DEGs associated with shattering. According to the above criteria, 980 DEGs were screened (Table S6).

### 2.6. Candidate Gene Analysis of qBTS1

**Figure 6:**
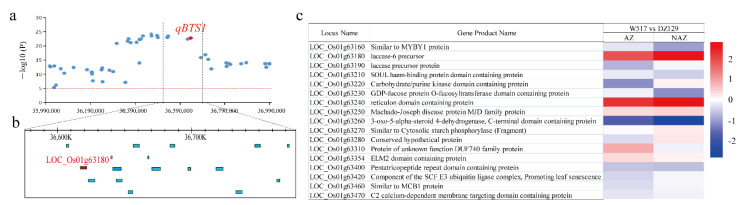
Identification of the candidate gene LOC_ Os01g63180: (**a**) Local Manhattan plot of BTS values surrounding the peak on chromosome 1. (**b**) Identification of candidate genes in the region of *qBTS1*. (**c**) The candidate genes of *qBTS1* and their expression patterns in the RNA-Seq results.

In our study, a major QTL, *qBTS1*, was repeatedly mapped. We chose to analyze the potential candidate genes in *qBTS1*, extending the region 100 kb upstream and 100 kb downstream (Figure 6a,b). Transposons and retrotransposons, however, were excluded. After analyzing the GWAS map and RNA-Seq results, we discovered 15 core differential genes, which were detected in both the GWAS and RNA-Seq. Among these 15 genes, we found two genes, *LOC_Os01g63260* and *LOC_Os01g63180,* that had significantly different expression (Figure 6c,d, Table S6). *LOC_Os01g63180* is annotated as a laccase precursor protein that degrades lignin-derived products. The *LOC_Os01g63260* gene is annotated as 3-oxo-5-alpha-steroid 4-dehydrogenase, and the C-terminal domain contains the protein involved in the reduction step in the biosynthesis of the plant steroid brassinolide. Therefore, further analysis by qRT–PCR revealed that the expression patterns of the candidate genes were similar to the transcriptome data (Figure S1). The loss of seed shattering in rice was previously reported to be related to lignin deposition [9,14], therefore the candidate gene *LOC_Os01g63180* was the focus of further analysis.

**Figure 7:**
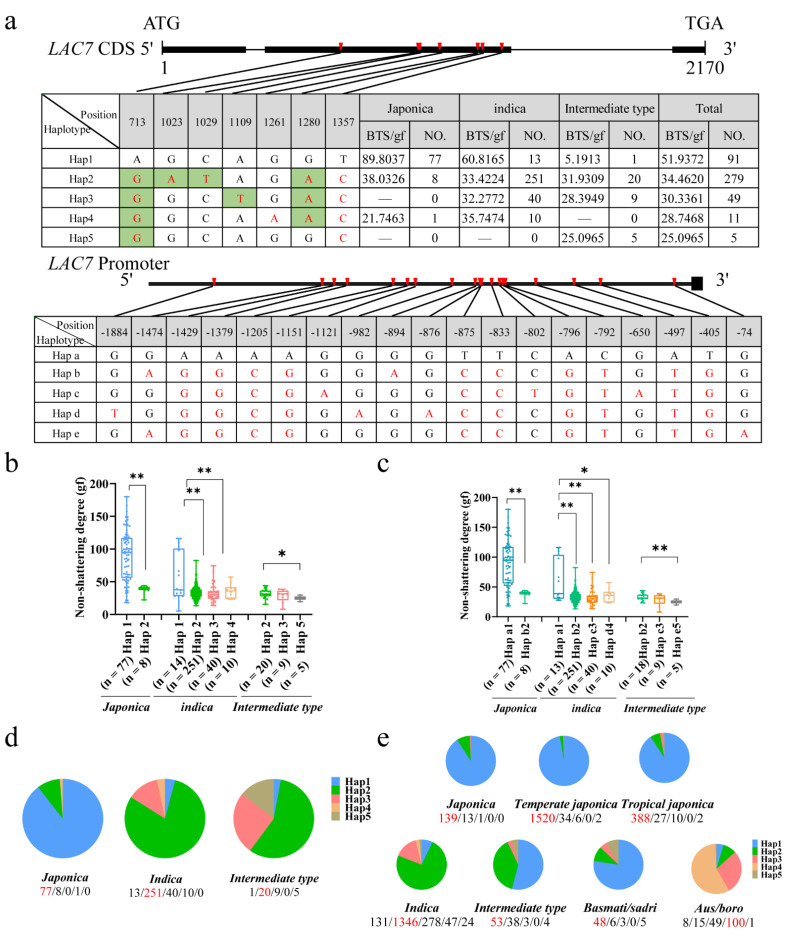
Candidate gene analysis of *qBTS1*: (**a**) Haplotype analysis of *LAC7* coding regions in 435 naturally occurring rice cultivars. Red letters symbolize different nucleotides, and green-marked letters symbolize nonsynonymous substitutions. (**b**) Haplotype analysis of the candidate gene *LAC7* in 435 natural rice cultivars (coding sequence (CDS) regions); ** indicates an extremely significant difference at the 1% level. * indicates a significant difference at the 5% level. (**c**) Haplotype analysis of the candidate gene *LAC7* in 431 natural rice cultivars (coding sequence (CDS) regions and promoter); ** indicates an extremely significant difference at the 1% level. * indicates a significant difference at the 5% level. (**d**) Distribution frequency of the five *LAC7* haplotypes in 435 naturally occurring rice cultivars. The numbers of cultivars with the haplotypes are given from left to right below the subpopulations. The haplotype with the largest number is highlighted in red. The same applies below. (**e**) Distribution frequency of the five *LAC7* haplotypes in a large sample of rice sequence data from MBKbase.

We carried out a gene-based haplotype-LOC_Os01g63180 (*LAC7*) analysis to confirm the association between the candidate gene *LAC7* and seed shattering by using resequencing data from 435 naturally occurring rice cultivars (Table S7). Within the coding region of *LAC7*, a total of seven SNPs were identified. Of the seven SNPs in the coding region, the five nucleotide substitutions, A713G, G1023A, C1029T, A1109T, and G1280A, caused amino acid conversions from Ile^213^ to Val^213^, Arg^316^ to Gln^316^, Thr^318^ to Met^318^, Asn^345^ to Tyr^345^, and Asp^402^ to Asn^402^, respectively, while the other two were synonymous substitutions (Figure 7a, Table S8). In addition, the haplotype analysis of the *LAC7* promoter region (2 kb) revealed co-inheritance with promoter and CDS haplotypes (Figure 7a,c, Table S9). The mutation of A713G in the CDS haplotype resulted in shattering changes (Figure 7a–c). We focused on nonsynonymous mutations based on polymorphisms, with 435 accessions being classified into five haplotypes (Figure 7d): 77 out of 86 (89.5%) japonica accessions carried Hap1, and few japonica rice accessions (9.3%) carried Hap 2; 13 out of 314 (4.1%) indica accessions carried Hap1, while 251 out of 314 (79.9%) carried Hap 2; 40 out of 314 (12.7%) carried Hap 3; and 10 out of 314 (3.2%) carried Hap 4. Most intermediate types carried Hap2, Hap3, and Hap5. Hap5 is associated with rare alleles and exists only in intermediate-type materials. In the analysis of the haplotype–phenotype associations, the material carrying Hap1 showed a loss of shattering phenotype; however, the material carrying other haplotypes showed shattering. In addition, there were extremely significant differences in the degree of shattering between Hap1-carrying materials and materials carrying other haplotypes (Figure 7b). All of these findings imply that *LAC7* is closely associated with rice seed shattering and may have contributed to the significant disparities between the indica and japonica subspecies’ seed-shattering phenotypes.

Additionally, we examined the distribution of the several haplotypes of the candidate gene *LAC7* in cultivated rice germplasms using a sizable sample of rice sequencing data from MBKbase (Figure 7e). The results showed that most of the japonica materials carried Hap1, specifically, 139 out of 153 (90.8%) japonica materials, 1520 out of 1562 (97.3%) temperate japonica materials, and 388 out of 427 (82.2%) japonica materials. Among the 1826 indica rice materials, 131 (7.2%) carried Hap1, and 1695 (92.8%) carried the remaining shattering haplotypes. The results showed that japonica cultivars, intermediate types, and Basmati/sadri had the highest frequency of Hap1, whereas indica cultivars and Aus/boro had the highest frequency of Hap2, Hap3, and Hap4. Overall, we concluded that *LAC7* was the most promising candidate gene for *qBTS1* by integrating GWAS mapping, transcriptomics, and haplotype analysis.

### 2.7. Candidate Gene Analysis of qBTS3

**Figure 8:**
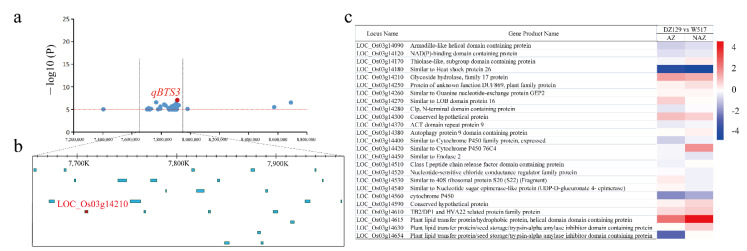
Identification of the candidate gene LOC_Os03g14210: (**a**) Local Manhattan plot of BTS values surrounding the peak on chromosome 3. (**b**) Identification of candidate genes in the region of *qBTS3*. (**c**) The candidate genes in *qBTS3* and their expression patterns in the RNA-Seq results.

The same approach described above identified 25 candidate genes in QTL *qBTS3* among the 64 candidate genes, and only *LOC_Os03g14210* was differentially expressed among the core shattering DEGs (Figure 8, Table S6). *LOC_Os03g14210* was annotated as glycosyl hydrolase family 17. The degradation of the AZ was previously reported to be related to hydrolases, therefore *LOC_Os03g14210* genes were further investigated. Further analysis by qRT–PCR revealed that the expression patterns of *LOC_Os03g14210* were similar to the transcriptome data (Figure S1).

**Figure 9:**
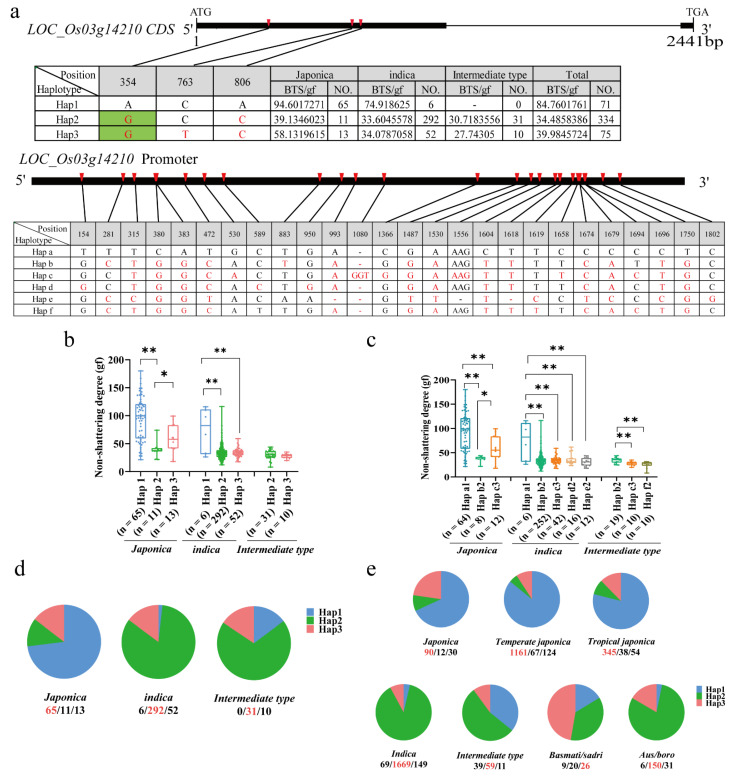
Candidate gene analysis of *qBTS3*: (**a**) Haplotype analysis of *LOC_Os03g14210* coding regions in 480 naturally occurring rice cultivars. Red letters symbolize different nucleotides, and green-marked letters symbolize nonsynonymous substitutions. (**b**) Haplotype analysis of the candidate gene *LOC_Os03g14210* in 480 natural rice cultivars (coding sequence (CDS) regions); ** indicates an extremely significant difference at the 1% level. * indicates a significant difference at the 5% level. (**c**) Haplotype analysis of the candidate gene *LOC_Os03g14210* in 451 natural rice cultivars (coding sequence (CDS) regions and promoter); ** indicates an extremely significant difference at the 1% level. * indicates a significant difference at the 5% level. (**d**) Distribution frequency of the five *LOC_Os03g14210* haplotypes in 480 naturally occurring rice cultivars. The numbers of the cultivars with the haplotypes are given from left to right below the subpopulations. The haplotype with the largest number is highlighted in red. The same applies below. (**e**) Distribution frequency of the five *LOC_Os03g14210* haplotypes in a large sample of rice sequence data from MBKbase.

We carried out a gene-based haplotype-*LOC_Os03g14210* analysis to confirm the association between the candidate gene *LOC_Os03g14210* and seed shattering by using resequencing data from 480 naturally occurring rice cultivars (Table S10). Within the coding region of *LOC_Os03g14210*, a total of three SNPs were identified. Of the three SNPs in the coding region, the one-nucleotide substitution, A354G, caused amino acid conversion from Gln^118^ to Arg^118^, while the other two were synonymous substitutions (Figure 9a, Table S11). Mutual analysis between the promoter region (2kb) and CDS haplotypes showed that haplotypes a1, b2, and c3 co-inherited in japonica, respectively; haplotypes a1 and c3 co-inherited in indica and intermediate types (Figure 9a,c Table S12). Interestingly, haplotypes b2, d2, e2, and f2 were present, with haplotypes b2 and f2 significantly regulating the difference in the shattering of the intermediate types (Figure 9c). We focused on the nonsynonymous mutations based on polymorphisms, with 480 accessions classified into 3 haplotypes (Figure 9d) (65 out of 89 (73.0%) japonica accessions carrying Hap1, 11 out of 89 (12.3%) carrying Hap2, 13 out of 89 (14.6%) carrying Hap3, 6 out of 330 (1.8%) indica accessions carrying Hap1, 292 out of 330 (88.5%) carrying Hap 2, and 52 out of 330 (15.8%) carrying Hap3). Most intermediate types carried Hap2 and Hap3. The results revealed that the material carrying Hap1 showed a loss of the shattering phenotype; however, the material carrying other haplotypes showed shattering. In indica rice, there were extremely significant differences in the degree of shattering between the materials carrying Hap1 and other haplotypes, while, in japonica rice, there were significant differences in the degree of shattering between the materials carrying Hap1 and Hap2 (Figure 9b). All of these findings imply that *LOC_Os03g14210* is closely associated with rice seed shattering and may have contributed to the significant disparities between the indica and japonica subspecies’ seed-shattering phenotypes.

We performed haplotype analysis of the *LOC_Os03g14210* gene in the MBKbase database by using the same method described above (Figure 9e). The results showed that most of the japonica materials carried Hap1, namely, 90 out of 132 (68.2%) japonica materials, 1161 out of 1352 (85.9%) temperate japonica materials, and 345 out of 437 (82.2%) japonica materials. Among the 1887 indica rice materials, 69 (3.7%) carried Hap1, and 1669 (88.4%) carried Hap2. The intermediate types, Basmati/sadri and Aus/boro, had the highest frequency of Hap2. Overall, we concluded that *LOC_Os03g14210* was the most promising candidate gene for *qBTS3* by integrating GWAS mapping, transcriptomics, and haplotype analysis.

## 3. Discussion

To date, most seed shattering genes have been cloned by map-based cloning, MutMap, and bulked segregant analysis [1,5,11,12,26,27]. Some mutant samples were used to perform whole-genome resequencing for the positional cloning of genes [28,29]; however, thus far, there have been few or no reports of candidate genes for positional cloning using GWAS. The GWAS population is rich in natural SNPs, and candidate genes can be mined without constructing an F2 population, which saves a great deal of personpower and time. In recent years, the use of GWASs to identify candidate genes associated with agronomic traits and stress has been widely reported [21,30,31]. In this study, 587 natural rice cultivated resources were used for a GWAS combined with RNA-Seq to mine candidate genes, which greatly saved time and labor.

Regarding, the effect of plant hormone signal transduction and the phenylpropane biosynthesis on seed shattering, Jiang LY et al. discovered that the RNA-Seq analysis of the young panicles of wild-type and *ssh1* mutant plants revealed DEG, and that DEG was primarily enriched in multiple biological processes, including the regulation of metabolic processes, the regulation of gene expression, transcription regulator activity, plant hormone signal transduction, and pathways involved in phenylpropanoid biosynthesis [12]. It was further proved that plant hormone signal transduction and phenylpropane biosynthesis pathways play an important role in regulating seed shattering in rice. In this study, transcriptome analysis using W517-DZ129 showed that AZ-specific DEGs of W517 were significantly enriched in plant hormone signal transduction, while AZ-specific DEGs of DZ129 included phenylpropanoid biosynthesis (Figure 5c,d). This study is consistent with previous reports. Transcriptome analysis results of weedy rice and cultivated rice showed that DEG was enriched in major metabolic and phenylpropane biosynthesis pathways, which further emphasized the role of abscisic acid (ABA) in the seed shattering of weedy rice [32].

New QTLs and potential seed shattering genes have been discovered.

Previous research has demonstrated that lignin concentration is a significant factor in both fruit dehiscence in Arabidopsis and seed shattering in rice and oil palm fruit abscission [9,33,34,35]. Because it catalyzes the last stage of monolignol polymerization, laccase is a crucial enzyme in the production of plant lignin [36,37]. Previous research showed that SNB modulates rice seed shattering by positively regulating the expression of *SH5* and *qSH1*, inhibiting lignin deposition in the AZ [9,12]. The interaction between *OSH15* and *SH5* may inhibit lignin deposition and affect seed shattering [14]. Floral organ shedding in Arabidopsis has been reportedly associated with a lignin molecular brace to support the cell wall [33]. Additionally, during the shedding of oil palm fruit, several candidate genes for the cell wall were discovered [35]. These genes shared similarities with those found to play functional roles during the shedding of Arabidopsis floral organs, anthers, siliques, or rice seeds, such as sequences with *ADPG1*, *PGAZ2*, *EGLAC7*, *EgMAN7*, *EgHSL1*, *CBSX1*, and *EgBEL1* [35]. Therefore, AZ development and lignin deposition have important effects on seed shattering [9,14,33]. In our study, based on combination of a GWAS and RNA-Seq results, we concluded that *LAC7* is an important candidate gene, and that *LAC7* is involved in the lignin catabolism process pathway with functions of scavenging and degrading lignin derivatives. Transcriptomic analysis showed that *LAC7* was significantly upregulated during high seed shattering (Figure 6c), degrading and removing lignin, and leading to a lignin reduction and the regulation of rice seed shattering. Previous studies have shown that hydrolase activity related genes are differentially expressed in Elymus sibiricus with high seed shattering and low seed shattering [38]. YQ Zhao et al. found that RAN-Seq of the AZ and NAZ of *Elymus nutans* and *E. sibiricus* identified three genes involved in polygalacturonase activity, five genes involved in hydrolase activity, and three genes involved in shattering [39]. In addition, hydrolases such as polygalacturonase and β-endoglucanase are activated in AZ cells, leading to the degradation of the intercellular layer and cell wall of isolated cells, and thereby facilitating shattering [40]. Additionally, little is known about the regulatory network mechanism through which the AL in rice cell wall degradation controls seed shattering. Another candidate gene identified in this study was *LOC_Os03g14210*, which is the glycoside hydrolase family 17, with hydrolase activity. This gene was significantly upregulated in high seed shattering and affected seed shattering in rice by degrading the AL of the AZ. Although it has been reported that hydrolase is related to the development and degradation of the AZ, the regulation effect of hydrolase on rice seed shattering needs further study. The identification of candidate genes in a rich population of natural variation makes up for the shortcomings of traditional mapping, and new valuable and important loci can be found from different perspectives. This provides a molecular basis for the further study of the molecular mechanism of seed shattering. In summary, we identified two candidate genes for the major QTLs *qBTS1* and *qBTS3*, encoding laccase precursor proteins with lignin scavenging and degradation functions and hydrolase family proteins with hydrolase activity, respectively. The identification and cloning of new candidate genes for rice seed shattering will provide new insights for the study of rice seed shattering.

## 4. Materials and Methods

### 4.1. Plant Materials

There were 587 different rice accessions in the mapping population. Most of them were from China, though there were also some from Japan, the Philippines, and other nations (Table S1). All plants were grown in the experimental field of South China Agricultural University (in Guangzhou, China) in the late season in 2020 and the early season in 2021. W517 has phenotypes such as seed shattering, awns, brown grains, and brown glumes and is a germplasm resource collected by the research group. Duzi 129 (DZ129) is a germplasm resource from Jilin City, Jilin Province, which has phenotypes such as awns, brown grains, and brown glumes but loss of seed shattering. The plants were planted and grown in the early season of 2021, as described above.

### 4.2. Genome-Wide Association Study

Library preparation was performed using the NEB Next^®^ Ultra DNA Library Prep Kit (NEB, Ipswich, MA, USA), followed by whole-genome sequencing at a 10× sequencing depth using an Illumina NovaSeq PE150 sequencer. After filtering out low-quality sequences, alignments were performed using the reference genome (MSU-RGAP 7.0) (http://rice.plantbiology.msu.edu/, accessed on 5 January 2021), and SNP detection was performed using the GATK software toolkit [41] and VCFtools software [42]. A total of 3,576,716 SNPs with a minimum allele frequency (MAF) greater than 5% and a deletion rate less than 20% were selected from these SNPs and then paired with 3K Rice Core SNPs [43], resulting in a total of 126,841 SNPs. The GWAS was conducted using a linear mixed model in Efficient Mixed-Model Association eXpedited (EMMAX) [44]. Only associations that exceeded the *p* value thresholds (<1 × 10^−5^) with clear peak-like signals within 200 kb of the leading SNP were considered association loci [45].

### 4.3. Phenotypic Evaluation

To assess the degree of shattering of different rice varieties at 30 days after heading (DAH), the breaking tensile strength (BTS) of the grain pedicel was evaluated using a digital force gauge [26]. For an individual plant, a total of 20 grains from two panicles were measured. The average value is used as an indicator to measure the seed shattering of the variety.

### 4.4. RNA Extraction, cDNA Library Construction, and RNA-seq

Total RNA isolation was performed using samples taken at 10 DAH from the AZ and nonabscission zone (NAZ) of W517 and DZ129. The AZ was less than 2 mm in length, and the NAZ included the rest of each pedicel [39]. RNA purity and quantity were evaluated using a NanoDrop 2000 spectrophotometer (Thermo Scientific, Waltham, MA, USA). RNA integrity was assessed using an Agilent 2100 Bioanalyzer (Agilent Technologies, Santa Clara, CA, USA). Then, the libraries were constructed using the TruSeq Stranded mRNA LT Sample Prep Kit (Illumina, San Diego, CA, USA) according to the manufacturer’s instructions. These libraries were sequenced using the Illumina HiSeq™ 2500 sequencing platform. Raw data (raw reads) in fastq format were first processed using Trimmomatic [46], and the low-quality reads were removed to obtain clean reads. The clean reads were mapped to MSU-RGAP 7.0 using HISAT2 [47]. Each gene’s FPKM value [48] was determined using Cufflinks [49], and HTSeq count [50] was used to determine each gene’s read counts. The DESeq (2012) R package was used to perform the differential expression analysis [51]. The cutoff for significantly different expression was established at a Q value < 0.05 and fold change >2. Based on the hypergeometric distribution, GO enrichment analysis and KEGG [52] pathway enrichment analysis of differentially expressed genes (DEGs) were carried out using R. transcriptome sequencing, and analysis were conducted by OE Biotech Co., Ltd. (Shanghai, China).

### 4.5. Candidate Gene Analysis

Using publicly available gene annotation datasets from RAP-DB (https://rapdb.dna.affrc.go.jp/, accessed on 5 May 2021) and MSU7 (Rice Genome Browser: http://rice.plantbiology.msu.edu, accessed on 5 May 2021), we searched for potential genes in the loci identified for the relevant characteristics. All the genes with annotations within 200 kb of peak SNPs were considered potential genes.

### 4.6. Quantitative Reverse Transcription PCR (qRT–PCR) Analysis

A portion of the total RNA used for the RNA-Seq investigation was needed to make cDNA for qRT–PCR. The AceQ qPCR SYBR Green Master Mix (Vazyme, Nanjing, Jiangsu, China) and reactions were run on ABI Step One Plus Real-Time PCR equipment in accordance with the manufacturer’s instructions (Applied Biosystems, Foster City, CA, USA). The actin gene (*LOC_Os01g71770*) was used as an internal control. Ten candidate genes were randomly selected from the transcriptome data to verify the accuracy of the transcriptome data, and the primers are shown in Table S13.

### 4.7. Scanning Electron Microscopy

Spikelet samples at 0 and 14 DAH were taken for scanning electron microscopy (SEM) to compare the morphological variations between the AZs (the rachilla beneath the floret) of W517 and DZ129. Dissections of at least three inflorescences from W517 and DZ129 were made.

### 4.8. Haplotype Analysis

The haplotypes of candidate genes were determined using the research group’s 587 different rice germplasm resequencing databases and Molecular Breeding Knowledgebase (MBKbase) [53]. Only haplotypes with more than ten accessions were considered.

### 4.9. Data Analysis

GraphPad Prism 5 software (GraphPad Software Inc., San Diego, CA, USA) was used to analyze the experimental data, and Student’s *t* test was used to detect significant differences between samples at the 5% and 1% levels of probability.

## Data Availability

All the RNA-seq data generated in this research was deposited in the Sequence Read Archive database (www.ncbi.nlm.nih.gov/geo, accessed on 6 May 2022) at NCBI (National Center for Biotechnology Information) under accession number: GSE211952. Reviewer link: https://www.ncbi.nlm.nih.gov/geo/query/acc.cgi?acc=GSE211952, accessed on 10 May 2022. The data sets supporting the results of this study are included in the manuscript and Supplementary Information Files. Rice seeds are available from the National Engineering Research Center of Plant Space Breeding, PR China.

## Supplementary Materials

The following supporting information can be downloaded at: https://www.mdpi.com/article/10.3390/ijms232314633/s1.
