# Supplementary figures and images for: RNA-Seq and Genome-Wide Association Studies Reveal Potential Genes for Rice Seed Shattering
Source: Int J Mol Sci. 2022 Nov 23;23(23):14633. doi: 10.3390/ijms232314633 (PMC9736558; doi:10.3390/ijms232314633)

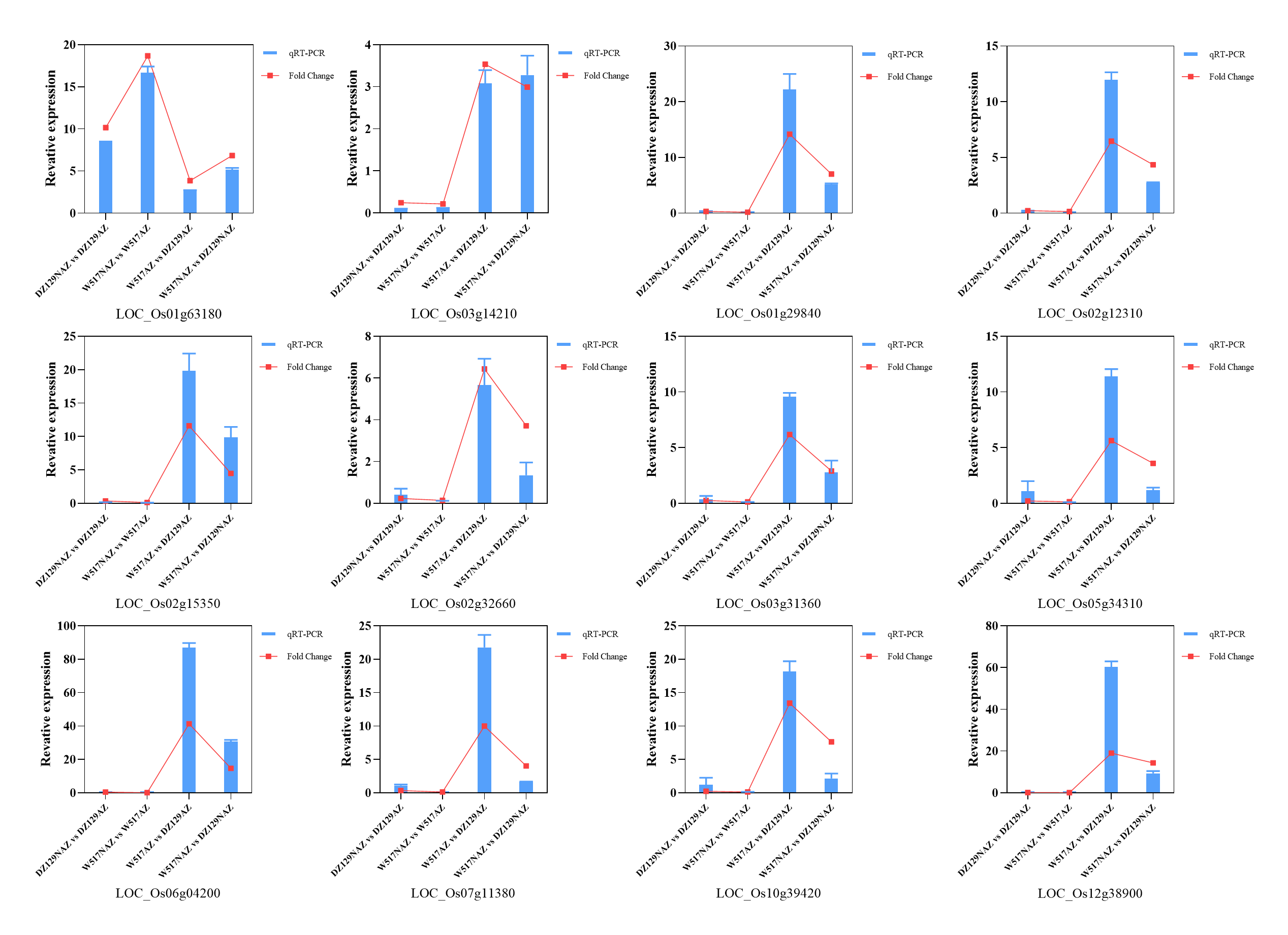

Supplement: Supplementary file 1 [file ijms-23-14633-s001.zip › ijms-2011518-supplementary/Supplementary Figure S1.tif]
